# Acceptance of Smart Technologies in Blended Learning: Perspectives of Chinese Medical Students

**DOI:** 10.3390/ijerph20032756

**Published:** 2023-02-03

**Authors:** Muhammad Azeem Ashraf, Nadia Shabnam, Samson Maekele Tsegay, Guoqin Huang

**Affiliations:** 1 Educational Science Research Institute, Hunan University, Changsha 410082, China; 2 Department of Health Professions Education, National University of Medical Sciences, Rawalpindi 46000, Pakistan; 3 School of Education and Social Care, Anglia Ruskin University, Cambridge CB1 1PT, UK

**Keywords:** smart technologies, blended learning, behavioural intention, medical education, higher education, China

## Abstract

Smart technologies are essential in improving higher education teaching and learning. The present study explores the factors that influence students’ behavioural intentions to adopt and use smart technologies in blended learning. Based on the Unified Theory of Acceptance and Use of Technology (UTAUT2) model, a survey of 305 students was conducted to collect data. A structural equation model was applied to analyse the data. The findings show that adopting smart technologies requires appropriate social context and organizational support. Moreover, the data indicated that performance expectancy, effort expectancy, social influence, hedonic motivation, and habit are vital in determining students’ behavioural intention to use smart technologies. However, facilitating conditions and price value were found to have no significant impact on the students’ behavioural intention to use smart technologies. The study contributes to a better understanding of the nexus of blended learning and smart technologies, thus improving students’ experiences in blended learning settings.

## 1. Introduction

The emergence of smart technologies has significantly affected people’s everyday life. Currently, mobile internet users in Mainland China have reached 1047 million, and the percentage accessing the internet via their mobile phones is 99.6% [1]. Moreover, online medical services have witnessed great development, accounting for 28.5% of all internet users in Mainland China [1]. Therefore, the Chinese government has set many policies and regulations to support the sustainable development of online medical services. For example, in January 2022, the Ministry of Industry and Information Technology, in collaboration with eight other departments, issued the 14th Five-Year Plan for the Development of Pharmaceutical Industry. This five-year plan aims to promote the development of healthcare services by integrating new and smart technologies into medical care in China [1,2]. Moreover, the development of smart technologies in health increases the availability of online resources so that individuals can easily access relevant information [2].

The term ‘smart technology’ refers to the use of different applications and tools in mobile and other wireless technologies that help individuals to achieve their desired objectives [3]. These technologies include educational technology tools (for example, laptops, projectors, smartphones, tablets, and internet of things), and applications (such as Google Meet, Microsoft Teams, Tencent Meeting, VooV Meeting, Kialo, Moodle, WeChat, Whatsapp, Zoom and other similar applications) that support the teaching and learning process [4,5]. They mostly influence how students are taught, which transforms the teaching and learning process and hence shapes knowledge transfer and sharing [6,7]. In this paper, we define smart technologies as a set of innovative technological tools to design an educational and developmental environment of a higher educational institution, aimed at ensuring the systemic realization of educational goals and comprehensive mastering of the content of professional training, as well as the introduction of appropriate forms, methods, techniques with significant developmental potential into the educational practice [8].

During the COVID-19 pandemic, many countries, including China, implemented lockdowns, quarantines, and other preventive measures in many sectors including higher education [9,10]. They replaced the teaching and learning process from face-to-face to online or blended modes. This shift in work and learning conditions created many opportunities for technological development, but it also caused many problems and challenges [9,10,11]. Considering COVID-19 and the development of new applications and tools, people were forced to use technologies to finish their tasks. This, therefore, increased the usage of smart technologies, which became an important and necessary tool for our daily life, including in the medical sciences. Although many studies have been conducted exploring the effects of technology from different perspectives in the past three years, there is still a need for further investigation to understand the issue from different parts of the world [12].

Similarly, the rapid and significant progress in developing smart technology has improved medical services, including teaching and learning in medical sciences in China [13,14]. Chinese higher education institutions have benefited from integrating smart technologies in their teaching and learning methods and shifting the traditional face-to-face learning methods to online or blended learning during the COVID-19 pandemic. This integration of technology into the traditional face-to-face teaching approach has made teaching and learning flexible and enjoyable and resulted in better learning outcomes [15,16]. The integration of smart technologies into education helped students continue their studies during the COVID-19 pandemic, using virtual classes from any part of the world [17]. Furthermore, smart technologies have helped students develop independent learning and foster critical thinking [16,18]. However, many factors such as pedagogical and technological skills and students’ access to vital resources affect the practice of blended learning, a method of education that combines online and face-to-face learning [9,19]. These challenges can be further divided into practical challenges and organizational challenges [12,18,19]. Practical challenges occur when individuals lack the necessary skills and knowledge to use smart technologies successfully. Whereas organizational challenges are associated with institutions’ inability to consider the current needs of students or hesitate to develop a facilitation culture to support their faculty and students to adopt new innovative methods in the teaching and learning process. For instance, a qualitative experimental study on blended learning suggested that insufficient teacher pedagogical skills and the intensive exam-oriented education system hinder the effective implementation of blended learning [18]. In addition, exposure to smart technologies may lead to unnecessary and harmful information and thus wastes students’ time on things that may cause psychological problems [7].

This study is part of a large project focusing on the development and improvement of blended learning in Chinese academia. Many results of this project using different research designs and approaches, such as qualitative experimental approaches [10,18], systematic review [19], and partial least squares-structural equation modelling (PLS-SEM) [20], have already been published in various journals, providing a better understanding of blended learning in higher education. This study is aimed at examining the factors that impact students’ willingness to use smart technologies in blended learning. There are numerous studies on the adoption of blended learning since it has started to become popular in higher education due to its benefits in supporting teachers to improve students’ learning and keep them engaged during the learning process [18]. However, very few studies are conducted focusing on the nexus of smart technologies and blended learning in medical classrooms. Since the restriction of COVID-19 has been relaxed, it is necessary to know if students who were forced to use smart technologies will continue using the technologies in the post-COVID era. In addition, the adoption of smart technologies in blended learning courses needs significant transformation not only in teachers’ and students’ technological skills, but also in their perception of education in general. These perceptions are vital to the success of smart technologies because they affect the methods of teaching and learning, teachers’ roles, and students’ class engagement and independent learning [21].

## 2. Research Model and Hypotheses

This study used the Unified Theory of Acceptance and Use of Technology (UTAUT2) model as its theoretical foundation. The UTAUT2 is one of the most intensive and advanced models of testing technology acceptance and adoption [22,23]. The UTAUT2 model has been extensively applied in academic research to examine the factors that impact individuals’ choices of adopting technologies in their studies and other parts of their life. This model is often compared to other models such as the Technology Acceptance Model (TAM), Social Cognitive Theory (SCT), Diffusion of Innovation (DOI), Theory of Planned Behaviour (TPB), Theory of Reasoned Action (TRA), and Motivation Model (MM). There is extensive literature available on the benefits and challenges of each model, as researchers use different models to conduct empirical studies. We selected the UTAUT2 model because it has better explanatory power regarding smart technology and its users [24,25]. Previous research in medical sciences has extensively applied UTAUT model to examine the acceptance of technology in healthcare institutions, and the majority of these studies found UTAUT more valid and beneficial in achieving better results in medical sciences [25,26,27].

Moreover, UTAUT2 is vital to study the methods, levels, and effects of accepting and understating new technologies in medical education [22,25]. Therefore, the model is relevant for this study considering the fact the study aims to exclusively investigate seven factors that may impact students’ behavioural intentions for accepting smart technologies in advancing medical knowledge. These seven factors are performance expectancy (PE), effort expectancy (EE), social influence (SI), facilitation conditions (FC), hedonic motivation (HM), price value (PV), and habit (HA).

### 2.1. Performance Expectancy (PE)

In this study, performance expectancy is the level at which students perceive that new smart technologies contribute to improving their performance [25]. The PE involves the perception regarding the effectiveness of smart technologies in enhancing individuals’ learning. Previous studies have confirmed that PE is a very influential factor for students using smart technologies in health education [3,26]. The following hypothesis is proposed for this study:

**H_1_.**  *Performance expectancy has a positive effect on students’ behavioural intention to use smart technologies.*

### 2.2. Effort Expectancy (EE)

Effort expectancy refers to the perception regarding the difficulty of a procedure or practice. This study applied EE as the difficulty or easiness of using smart technologies in medical education. Previous studies indicated that individuals’ commitment to adopting new technologies depends on their ease of use [3,27]. Thus, EE is another influential factor in an individual’s behavioural intention to use technology. Therefore, the following hypothesis is proposed:

**H_2_.**  *Effort expectancy has a positive effect on students’ behavioural intention to use smart technologies.*

### 2.3. Social Influence (SI)

Social influence is defined as the impact of other people’s beliefs, interpretations, and practices on adopting smart technologies. Many studies have identified that the views and practices of colleagues, peers, teachers, and friends affect an individual’s technical identity in using technology. For example, Alrawashdeh et al. [28] found that individuals are influenced by their cohorts’ views and experiences of using technology for learning. Other studies also showed the substantial relationship between social influence and students’ behavioural intentions to accept technologies [27]. In line with this, the following hypothesis is proposed:

**H_3_.**  *Social influence has a positive effect on students’ behavioural intention to use smart technologies.*

### 2.4. Facilitation Condition (FC)

The facilitation condition is defined as the level of influence that support and assistance provide to individuals to apply technology. Previous studies showed that using technology requires individuals to reach a certain level of relevant knowledge [27,28]. Professional training and special assistance improve the willingness of individuals to use technology. The following hypotheses are proposed:

**H_4_.**  *Facilitation condition positively affects students’ behavioural intention to use smart technologies.*

**H_5_.**  *Facilitation condition positively affects students’ actual behaviour to use smart technologies.*

### 2.5. Hedonic Motivation (HM)

Hedonic motivation means the level of influence due to individuals’ pleasure in using technology. It is characterized by students’ satisfaction and enjoyable experience towards using smart technologies in their learning. Previous studies showed that the happiness emerging from using technology could play a considerable role in deciding the adoption of new technologies [29]. However, very few studies have included this variable in evaluating their models. The results of these studies suggest that hedonic elements of educational resources are essential in improving students’ learning experience. Thus, the following hypothesis is proposed:

**H_6_.**  *Hedonic motivation has a positive effect on students’ behavioural intention to use smart technologies.*

### 2.6. Price Value (PV)

The price value is the perceived value of using technologies, which is often referred to as individuals’ cognitive trade-off between the perceived benefits of technologies and the monetary cost of using them. The individuals’ positive perception of the benefits of using technology influences their intentions to bear the cost of the technology used [29]. This factor received very little attention from researchers in education, mostly due to its concept of good value for money. However, this study used this factor as the value associated with students’ learning gained from smart technologies, which determines the perceived value of these technologies in learning. Even though students might not have to bear any monetary cost, they devote time and effort to benefit from smart technologies. Therefore, students’ positive perception of using smart technologies for learning is expected to consider spending more time and effort in order to effectively use them. Thus, the following hypothesis is proposed:

**H_7_.**  *Price value has a positive effect on students’ behavioural intention to use smart technologies.*

### 2.7. Habit

The habit is identified as the extent to which individuals consider the behaviour and performance to be automatic. Various approaches established by previous studies indicate that habit influences the intention to use new technologies, and it positively impacts students’ intention to use new technologies in their learning [29]. Hence, the following hypothesis is proposed:

**H_8_.**  *Habit has a positive effect on students’ behavioural intention to use smart technologies.*

### 2.8. Behavioural Intention

Behavioural intention is defined as individuals’ willingness to use a particular technology to perform different tasks. It identifies the intensity of individuals’ commitment to engage in specific actions that result in the actual behaviour. For example, our previous study revealed that teachers in China appreciate the use of blended learning, but they did not intend to adopt it in their teaching due to limited pedagogical skills and the exam-oriented education system in China [18]. However, the current study was conducted during the COVID-19 pandemic, in which the use of online or blended learning was not an option but a necessity. Yet many studies indicate that behavioural intentions to use technologies substantially affect actual technology use [27,30]. In line with this, this study assumes that behavioural intention to use technology can have a significant impact on individuals’ actual use of smart technologies. The following hypothesis is proposed:

**H_9_.**  *Students’ behavioural intention to use blended learning has a positive influence on the actual use of smart technologies.*

## 3. Research Methods

### 3.1. Population and Participants

This research employed a quantitative approach to collect data from students enrolled in medical sciences at universities in the Hunan province of China. The universities in Hunan province, similar to other universities in China, have been teaching using blended learning since the start of COVID-19 in early 2020. However, the intensity and depth of blending online classes with in-person classes was determined based on the severity of the pandemic in the region. In this learning mode, Tencent Meeting, WeChat, QQ, universities’ learning management systems and other applications were commonly used by teachers and students in blended learning. Online questionnaires were sent to the students enrolled in medical sciences at universities located in Hunan province in March 2022, and 320 completed questionnaires were received by the end of April. However, 305 questionnaires were found valid and used for the study. Considering the context of the study and the research method used, the sample size is considered sufficient and representative. The study followed proper ethical procedures throughout the research. The participants were informed about the aim and purpose of the survey and were asked to sign a consent form before participating in the study. Ethical approval for the study was obtained from Hunan University.

### 3.2. Instrument Development

**Figure 1:**
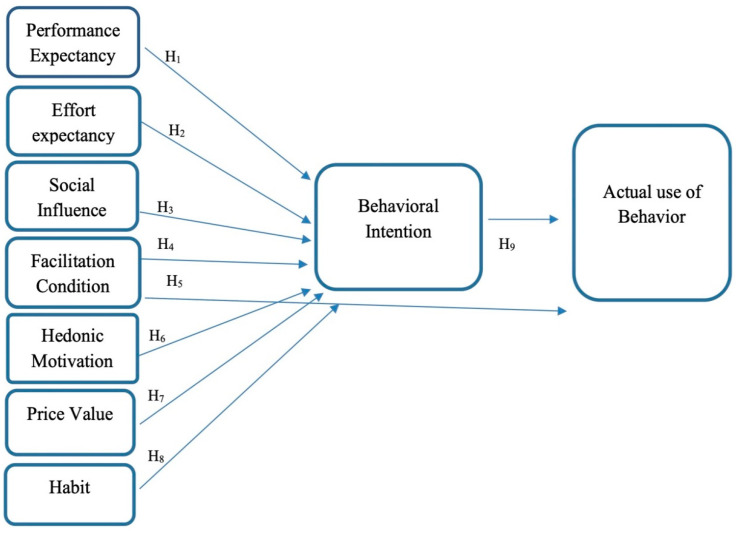
Proposed Theoretical Research Method Adapted from UTAUT2.

To collect the data, a questionnaire consisting of demographic information and the use of smart technologies in blended learning was developed based on previous studies [27,30,31,32]. The demographic information included age, gender, level of education, and previous experience (before COVID-19) with blended learning. The second part consists of 35 items to measure the nine constructs of the research model (see Figure 1). The questionnaire items were designed based on the UTAUT2 framework while multiple items were used to measure each construct. Among these constructs, PE, EE, and SI have five items each; HM and HT have four items each; FC, PV, and actual use of behaviour have three items each; and BI has two items. A 5-point Likert scale consisting of five answer options ranging from “strongly disagree” (number 1) to “strongly agree” (number 5) was used to score questionnaire responses to quantify the constructs.

### 3.3. Statistical Technique

The collected data were analysed using SPSS (version 27, SPSS Inc., Chicago, IL, USA) and AMOS (version 27, IBM, Armonk, NY, USA) software. Pearson’s correlation test was performed to extract the correlations between variables and constructs. Initially, the descriptive analysis was performed by SPSS and then the Structural Equation Model (SEM) was used to estimate the path of the hypothesized constructs. The significant level was set at *p* ≤ 0.05. The nine constructs and 35 items measuring these constructs in the proposed model are presented in the Appendix (see Appendix A). Two types of validity measures such as convergent validity and discriminant validity were used to check the validity of model constructs. The convergent validity was assessed using Factor Loadings (FL), Cronbach’s Alpha (CA), Composite Reliability (CR) and Average Variance Extracted (AVE). The acceptable levels were found to be greater than 0.70 for FL, CA, and CR, and above 0.50 for AVE. The discriminant validity was assessed by comparing the correlation coefficients between the constructs and the square root of AVE.

## 4. Data Analysis and Results

### 4.1. Descriptive Analysis

The mean values of almost all the items (see Appendix A) were above the mid-point of 3.5, suggesting that the respondents had generally given positive responses to the measured items. The standard deviations ranged from 0.738 to 0.977, showing a narrow spread around the mean. The socio-demographic characteristics of the respondents are presented in Table 1, which shows that out of 305 respondents, 48.2% were males and 51.8% were females. Regarding age distribution, 64.6% were less than 23 years old, approximately 31% were 24–28 years old, and almost 5% were more than 29 years old. Respondents’ levels of education varied, showing that 62.3% were undergraduate students; 33.8% were master’s degree students, whereas the rest (3.9%) were doctoral students.

### 4.2. Measurement Model Evaluation

The measurement model was evaluated using the internal reliability and validity of the measures and endorsing their reliability, convergent validity, and discriminant validity. Table 2 shows that the estimated construct loadings range from 0.681 to 0.960, which is higher than the recommended levels [33]. Construct reliability indicates how well a construct is measured by its items and can be measured based on Cronbach’s alpha and CR. The Cronbach’s alpha values ranged from 0.74 for SI to 0.87 for EE, and CR values ranged from 0.761 for SI to 0.89 for EE. For both measures, all constructs exceeded the recommended cut-off of 0.7 [33,34], thereby suggesting high internal reliability and confirming that all measures are rigorous in terms of their reliability. Convergent validity was measured by checking the standardized factor loadings and average variance extracted (AVE) following Fornell and Larcker’s recommendation [34]. Convergent validity is verified when (i) all measurement items are greater than 0.70, (ii) composite reliability is above 0.70, and (iii) average variance extracted (AVE) tops 0.50 [33,34]. In this study, these requirements were all achieved (see Table 2). Therefore, the results offered strong confirmation of convergent validity.

To evaluate discriminant validity, the square roots of the AVEs were compared with the inter-construct correlations to ensure that each factor was different or uncorrelated. Table 3 shows that all correlation coefficients between factors in the model were below the square root of the AVEs, meaning that the constructs were unlike each other. These results showed that the questionnaire had very good discriminant validity. The results given in Table 3 also demonstrated that HA had the strongest positive association with students’ behavioural intention to use blended learning. Likewise, a statistically significant association also was found between PE and students’ BI to use blended learning. These results (Table 3) demonstrated the nonexistence of multicollinearity in the research because a very high correlation was not observed between the model variables [27].

### 4.3. Structure Model

**Figure 2:**
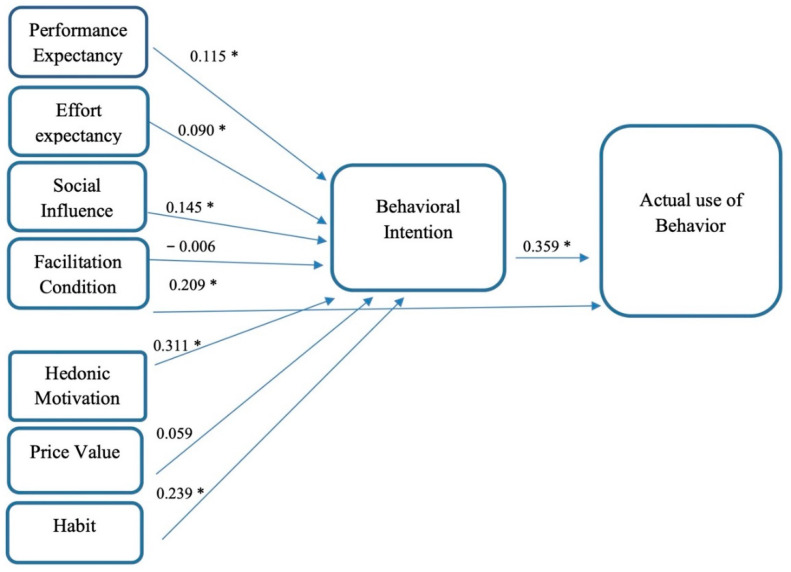
Results of Structural Model.

After establishing good convergent and discriminant validity, the next step was to assess the structural model to test the proposed relationships. It was judged by examining the standardised beta coefficients and t-values of the hypothesised model. Factors such as PE, EE, SI, FC, HM, PV and HA were entered as independent variables, while behavioural intention and actual usage were entered as dependent variables in the model. The R^2^ values of the behavioural intention and actual usage were 0.69 and 0.56, respectively, demonstrating that all independent variables accounted for 69% of the total variance in students’ behavioural intention to use blended learning. Indeed, their behavioural intention also accounted for 56% of the total variance in their actual usage of this approach. These results signified sufficient model fit between the posited research model and the empirical data. The results of the structural equation model are given in Table 4 and illustrated in Figure 2. In detail, H1 determined whether PE has a significant positive effect on students’ behavioural intention to use blended learning. The findings show that performance expectancy was a significant predictor of students’ behavioural intention in this regard (β1 = 0.115, *t*-value = 2.058, *p* < 0.05), thereby endorsing H1. Similarly, the rest of the indicators which had positive and significant effects on the students’ behavioural intention to use blended learning include H2 = EE (β = 0.090, *p* ≤ 0.05), H3 = SI (β = 0.145, *p* ≤ 0.05), H5 = FC (β = 0.239, *p* ≤ 0.05), H6 = HM (β = 0.311, *p* ≤ 0.05), and H8= HA (β = 0.239, *p* ≤ 0.05). The SEM findings disclosed that FC had an insignificant effect on students’ behavioural intention, in this respect β = −0.006, *p* > 0.05, thus rejecting H4. PV also had an insignificant effect on the students’ use of blended learning. Further, the students’ behavioural intention to use blended learning had a significantly positive effect on the actual use of blended learning (β = 0.359, *p* ≤ 0.05).

## 5. Discussion

The purpose of this study was to explore the main factors that influence the acceptance of smart technologies in blended learning in medical education in Chinese higher education. The conceptual framework is based on UTAUT2 to find the behavioural intention of students towards using smart technologies in blended learning courses. The results showed that students’ behavioural intention to use smart technologies was significantly influenced by performance expectancy, effort expectancy, habit, and hedonic motivation. On the other hand, contrary to our expectations, facilitating conditions and price value did not influence students’ behavioural intentions to use smart technologies.

The empirical results demonstrated that performance expectancy was a significant determinant of behavioural intention to use smart technologies. It is, therefore, believed that students who found the system useful in their learning process will be more willing to adopt new smart technologies during blended learning. Hence, in order to attract more users of smart technologies, instructors should improve the content quality of their resources by providing adequate and conversant content that can fit the students’ needs. These results support previous studies conducted by various scholars [27,30]. Other studies, including Abdekhoda et al. [31] and Tarhini et al. [35], also found the direct effect of PE on the students’ BI to use e-learning. Therefore, smart technologies in blended learning in medical education are essential and valuable. This technique enhances their productivity and strengthens their proficiency in using the technology for learning and engaging in other technology-based activities. This finding is also consistent with the study of Suki and Suki [27].

The findings of this study further revealed that effort expectancy positively influenced the students’ behavioural intention to use smart technologies in blended learning. These findings are in line with those reported in earlier studies such as Alrawashdeh et al. [28], Bashirian et al. [36], Abdekhoda et al. [31], and Tarhini et al. [35], which showed that effort expectancy had a significant and positive effect on the use of technologies. Alalwan et al. [37] also reported that effort expectancy considerably influences the willingness to use online learning. This demonstrates that practical training should be directed to less skilled individuals, instead of those who had some training before. Moreover, system designers should provide a system that promotes ease of online learning by collecting feedback from end-users, teachers and students. With such improvements, the teaching-learning process could be easier, participatory and enjoyable in blended learning.

Studies have shown that peers’ and instructors’ opinions can affect others’ beliefs and intentions about using technology and things associated with it [35]. Similarly, the findings of this current study revealed a positive relationship between social influence and behavioural intention to use smart technologies in blended learning. The results in this study corroborated the earlier studies [30,38,39,40].

The other two significant factors that positively affected students’ behavioural intention to use smart technologies in blended learning are hedonic motivation and habits. The results in this study indicated that these two factors are critical determinants of behavioural intention, which are in line with the findings of many other researchers [30,35,39,40,41,42], and consistent with UTAUT2 methodology. In other words, pleasant learning experiences are important factors in using smart technologies during blended learning. On the other hand, a user-friendly environment and digital content have a significant impact on producing pleasurable learning experiences [43]. This suggests that educational designers should pay special attention to these features as they affect students’ learning and academic progress. If students are happy with using online learning, they are more likely to advance their independent learning skills. Hence, hedonic motivation and habits are critical in expanding the scope and generalizability of UTAUT2, not only in the e-learning setting but also in the blended learning atmosphere.

On the other hand, the two constructs (i.e., facilitating conditions and price value) were found to have no significant impact on the students’ behavioural intention to use smart technologies during blended learning. This result consolidated the findings of the studies conducted by Abdekhoda et al. [31], Tarhini et al. [35] and Azizi et al. [30], but is in contrast with other studies [27,40,42,44,45].

Finally, this study suggests that behavioural intention positively affects students’ actual use of blended learning. This also correlates with studies that argue that the actual use of blended learning depends on the behavioural intention to use the teaching approach [26,29,30,46].

## 6. Conclusions

Using the UTAUT2 framework, this study examined the factors affecting the acceptance of smart technologies in blended learning courses in medical education at Chinese universities. The COVID-19 pandemic forced universities to adopt a blended learning approach, and it was the first time for the majority of students to attend blended learning courses. This study suggested that providing a social context and organizational support and changing the students’ psychological attitudes toward new learning approaches are essential steps in successfully implementing new smart technologies in blended learning. Moreover, the results demonstrated that the model designed based on UTAUT2 was found to be suitable for determining the factors influencing the use of smart technologies in blended learning in medical education. The performance expectancy played a significant role in determining the students’ behavioural intention to use smart technologies in blended learning in China, and this variable was followed by effort expectancy, social influence, hedonic motivation and habit. This outcome is consistent with studies conducted by other researchers [30,31,35].

This study has some theoretical and practical implications. In theory, this study provides support and explanation of the UTAUT2 framework in educational settings. In practice, this study presents the important factors affecting students’ choices of using smart technologies in their learning. Given the fact that almost every university student has a smartphone in China, the implementation of smart technologies could be easy. Thus, more studies are required to advance knowledge, such as in different cultures, majors, and teaching methods. The study has some limitations. First, the study used a self-reporting scale to collect the data, which may lead to some errors. The number of completed questionnaires is also low compared to the number of universities (and medical students) in Hunan province. Second, this study did not test any mediating factor that may affect the relationship between factors and students’ intention to use smart technologies. Third, it included medical students, only. Therefore, future studies that incorporate different mediating factors are required to improve and better understand the use of smart technologies among medical students. In addition, comparative and experimental studies on the use and effects of smart technologies on students learning are vital to understand the situation from different perspectives and disciplines.

## Figures and Tables

**Table 1:** Socio-demographic Characteristics of respondents.

Variable	Frequency	Percentage
Gender		
Male	147	48.2
Female	158	51.8
Age Groups in Years		
Below 18	10	3.3
19–23	187	61.3
24–28	93	30.5
29–33	7	2.3
34 and above	8	2.6
Education Level		
Undergraduate	190	62.3
Masters	103	33.8
Doctoral	12	3.9
Experience of Online Learning (pre-COVID-19)		
Yes	235	77
No	70	23

**Table 2:** Measurement Model Results.

Constructs	Items	Factor Loading	Cronbach’s Alpha α	CR	AVE
Performance Expectancy	5	0.664–0.802	0.851	0.884	0.658
Effort Expectancy	5	0.739–0.843	0.876	0.850	0.589
Social Influence	5	0.505–0.669	0.740	0.884	0.656
Facilitation Condition	4	0.563–0.900	0.708	0.853	0.593
Hedonic Motivation	4	0.589–0.806	0.846	0.870	0.572
Price Value	3	0.604–0.699	0.813	0.842	0.828
Habit	4	0.647–0.822	0.778	0.902	0.692
Behavioural Intention	3	0.538–0.639	0.813	0.935	0.718
Actual Use of Behaviour	4	0.604–0.846	0.838	0.752	0.892

**Table 3:** Inter-Construct Correlations.

Constructs	PE	EE	SI	FC	HM	PV	HA	BI	UB
**PE**	**0.81**								
**EE**	0.226 *	**0.77**							
**SI**	0.228 *	0.198 *	**0.81**						
**FC**	0.228 *	0.173 *	0.309 **	**0.77**					
**HM**	0.123 *	0.007 *	0.240 *	0.293 *	**0.76**				
**PV**	0.331 *	0.136 *	0.386 *	0.342 **	0.373 *	**0.91**			
**HA**	0.268 *	0.172 **	0.240 *	0.326 **	0.287 *	0.380 *	**0.83**		
**BI**	0.241 *	0.308 **	0.345 **	0.332 *	0.401 *	0.289 **	0.480 *	**0.85**	
**UB**	0.340 *	0.271 **	0.450 **	0.350 *	0.269 *	0.232 **	0.448 *	0.502 *	**0.94**

Note: * Correlation is significant at the 0.05 level (two-tailed), and ** correlation is significant at the 0.01 level (two-tailed). Diagonal elements in bold show the square root of AVE.

**Table 4:** Relationship of students’ behavioural intention to use smart technologies in blended learning.

Hypothesis	Hypothesised Path	Estimates	S.E	*t*-Value
H_1_	PE→BI	0.115 *	0.056	2.058
H_2_	EE→BI	0.090 *	0.033	2.722
H_3_	SI→BI	0.145 *	0.054	2.658
H_4_	FC→BI	−0.006	0.048	−0.134
H_5_	FC→AU	0.209 *	0.041	2.576
H_6_	HM→BI	0.311 *	0.061	5.117
H_7_	PV→BI	0.059	0.056	1.052
H_8_	HA→BI	0.239 *	0.054	4.445
H_9_	BI→AU	0.359 *	0.049	3.425

Notes: * *p* < 0.05; S.E. = Standard error.

## Data Availability

The data supporting the findings of this study are not publicly available due to ethical restrictions.

## Appendix A

**Table A1:** Measurement Items.

Constructs	Items	Mean	SD
Performance Expectancy	Using the online learning would improve my learning performance.	3.36	0.770
	Using online learning increases my chances of achieving learn that are important to me	3.60	0.857
	Using the online learning would allow me to accomplish learning tasks more quickly	3.39	0.867
	Using the online learning would enhance my effectiveness in learning.	3.30	0.915
	Using the e-learning system makes it easier to learn course content.	3.48	0.847
Effort Expectancy	Adopting the method of the online learning system is easy for me	3.58	0.831
	My interaction with the online learning system is clear and understandable.	3.50	0.836
	It is easy for me to become skilful at using the online learning system.	3.63	0.891
	I find online learning easy to use.	3.73	0.839
	I would find it easy to get the online learning to do what I want it to do.	3.72	0.850
Social Influence	People who are important to me think that I should adopt the online learning system.	3.23	0.824
	People who influence my behaviour think that I should use the online learning system	3.26	0.836
	My instructors thinks that I should participate in the online learning activities.	3.40	0.853
	The opinion of non-academic groups (e.g., friends and family) is important to me.	3.39	0.886
	In general, the university has supported the use of online learning activities.	3.77	0.810
Facilitation Condition	I have the resources necessary to use the online learning system.	3.68	0.792
	I have the information necessary to use the online learning system.	3.64	0.749
	A specific person or team is available for support with online learning difficulties.	3.44	0.905
	WBT is not compatible with other systems I use.	2.92	0.997
Hedonic Motivation	Computers and online learning services make learning more interesting.	3.63	0.809
	Learning about using computers and online services is fun.	3.63	0.764
	I like using computers.	3.54	0.899
	I look forward to those aspects of my learning activities that require me to use computers.	3.54	0.807
Price Value	Online learning is reasonably priced.	3.26	0.866
	Online learning is a good value for the money.	3.37	0.841
	At the current price, online learning provides good value.	3.35	0.883
Habit	The use of the internet and the online learning system has become a habit for me.	3.45	0.854
	I am addicted to using the internet and the online learning system for educational purposes.	3.28	0.919
	I must use the internet and online learning in my learning activities.	3.15	0.977
	Using the internet and online learning system has become natural to me.	3.59	0.802
Behavioural Intention	I intend to use online learning in the future.	3.41	0.885
	I am sure I will use online learning in the future.	3.68	0.758
	I predict I will take online learning courses in the future.	3.75	0.775
Actual Use of Behaviour	Online learning makes work more fascinating.	3.53	0.739
	Using online learning is a good idea.	3.82	0.738
	Working with online learning management systems is a pleasure.	3.56	0.825
	I like working with online learning	3.56	0.829
